# Gastrointestinal health and nutritional strategies in autism spectrum disorder

**DOI:** 10.1007/s00535-025-02269-1

**Published:** 2025-06-18

**Authors:** Yuqi Wu, Oscar Wing Ho Wong, Sizhe Chen, Siew Chien Ng, Qi Su, Francis Ka Leung Chan

**Affiliations:** 1 Microbiota I-Center (MagIC), 10/F, Building 17W, 17 Science Park West Avenue, Hong Kong Science Park, Hong Kong SAR, China; 2 Department of Medicine and Therapeutics, The Chinese University of Hong Kong, Hong Kong SAR, China; 3 Department of Psychiatry, The Chinese University of Hong Kong, Hong Kong SAR, China; 4 The D.H. Chen Foundation Hub of Advanced Technology for Child Health (HATCH), The Chinese University of Hong Kong, Hong Kong SAR, China; 5 Li Ka Shing Institute of Health Sciences, State Key Laboratory of Digestive Disease, Institute of Digestive Disease, The Chinese University of Hong Kong, Hong Kong SAR, China; 6 Centre for Gut Microbiota Research, The Chinese University of Hong Kong, Hong Kong SAR, China; 7 Li Ka Shing Institute of Health Sciences, State Key Laboratory of Digestive Disease, Institute of Digestive Disease, The Chinese University of Hong Kong, Hong Kong SAR, China

**Keywords:** Gastrointestinal symptoms, Autism, Nutritional management, Precise nutrition, Clinical practice

## Abstract

Beyond the hallmark social and sensory difficulties in autism spectrum disorder (ASD), the comorbid gastrointestinal (GI) conditions and their potential link to the severity of core symptoms require clinical attention. Although evidence indicates that autistic children face a greater risk of GI disorders and require more intensive nutritional management compared to neurotypical peers, standard guidelines for managing GI symptoms in this population remain lacking. This review seeks to pinpoint critical considerations for the implementation of nutrition-based strategies aimed at addressing GI dysfunction in individuals with ASD. By emphasizing clinical translation and the mechanistic understanding of these strategies, it highlights the importance of restoring gut homeostasis as a pathway to improve functional independence and overall well-being. Furthermore, we outline priorities for clinical research aimed at developing evidence-based nutritional recommendations to support GI health in autistic individuals, emphasizing personalized and population-specific needs.

## Introduction

Autism spectrum disorder (ASD) represents a clinically heterogeneous neurodevelopmental condition that affects 1 out of 68 children, according to the latest epidemiological data [1]. Growing evidence demonstrates a common comorbidity between ASD and gastrointestinal (GI) dysfunction, with symptoms onset typically occurring during early developmental stages. It is estimated that children with ASD exhibit a fourfold increase risk (prevalence range: 9–91%) [2–4] of developing GI symptoms compared to their neurotypical peers. Common issues include constipation, diarrhea, bloating, abdominal pain, and other digestive problems like malabsorption and digestive enzymes deficiencies. Among these, constipation stands out as the most prevalent manifestation, demonstrating the increased likelihood with more severe verbal and social impairment [3]. Importantly, autistic children grappling with GI symptoms are also at a higher risk of experiencing severe comorbidities such as anxiety, depression, intellectual disabilities, insomnia, food allergies, and nutritional deficiencies [5, 6]. These findings suggest a vicious circle entangled by GI issues and brain dysfunction, highlighting the need for targeted interventions within autistic populations.

While the reasons behind the high comorbidity of GI symptoms in individuals with ASD are not entirely understood [7, 8], gut dysbiosis might be a culprit within the postulated disrupted gut–brain axis. In the autistic population, a prevalent dysbiosis—characterized by an altered ratio of pathogenic to commensal gut microbiota—is associated with intestinal inflammation, subsequent neuro-immune dysregulation, and sensory sensitivities. These interconnected mechanisms can profoundly compromise GI health [8]. Moreover, maladaptive eating behaviors that are frequently observed in ASD—such as food neophobia, restrained eating, and hypersensitivity to textures—can result in nutritional imbalances, further exacerbating malabsorption, constipation, and diarrhea [8]. However, these clinical manifestations commonly lead to misconceptions among parents or caregivers of autistic children. Behaviors such as dietary refusal or withdrawal from dietary interventions should not be mistakenly ascribed to behavioral noncompliance, but rather recognized as potential indicators of underlying medical conditions, particularly digestive enzyme deficiencies [8, 9]. Failure to properly identify these physiological etiologies may result in inappropriate nutritional treatments that not only prove ineffective in addressing malnutrition but could also cause developmental delays and side effects in this vulnerable population. More importantly, the increased use of medications and hypersensitive immune responses in ASD may adversely affect GI health while addressing ASD-related symptoms [2]. Therefore, autistic individuals suffering from GI symptoms may need a multidisciplinary approach to manage the concurrent nutritional and behavioral symptoms associated [10, 11]. Despite this clinical need, current evidence-based guidelines lack standardized protocols for the nutritional management of GI symptoms in ASD populations, highlighting a critical gap in therapeutic recommendations [2, 12].

In this review, we provide an updated overview of nutritional strategies for improving GI health in ASD, along with a comprehensive summary of the current clinical evidence. By dissecting the potential mechanisms by which nutritional approaches can ameliorate GI symptoms in the autistic populations, we highlight the individual variations and the potential adverse effects in response to these treatments. Even though there is no established standard for nutritional management of GI symptoms in ASD, this review offers valuable insights into the diet–gut microbiome interactions, highlighting their potential as a critical foundation for developing future nutritional strategies in ASD.

## Nutritional strategies to enhance GI health in ASD

Given the findings that autistic children typically have restricted diets and the strong links between ASD and nutritional status, nutritional therapy is increasingly recognized as a vital aspect of managing ASD with primary motivation over the years [12, 13]. Unlike conventional pharmacotherapy, which often carries significant side effects, nutritional interventions demonstrate superior safety profiles and provide holistic benefits that extend not only to GI improvement but also to mental health [14, 15].

Several dietary interventions have been investigated in ASD, including: gluten- and casein-free (GF/CF) diet [16, 17], ketogenic diet (KGD) (standard or modified gluten-free versions) [16, 18], and low-FODMAP diets [19]. Because poorly absorbed carbohydrates are especially problematic for sensitive individuals with ASD, low-FODMAP diets, which limit fermentable oligosaccharides, disaccharides, monosaccharides, and polyols, were recommended to alleviate GI distress [19]. However, the efficacy of these treatments is inconclusive, and side effects have been reported [20–22]. On the other hand, emerging evidence suggests that nutritional supplementation can be a therapeutic approach for ASD managements. Key interventions under investigation include: multivitamins (especially vitamin D) [23], multi-mineral (especially single trace element of zinc/iron) [23, 24], polyunsaturated fatty acids (PUFA) [23], antioxidants (flavonoids, polyphenols, sulforaphane, cacao, and cysteine-rich whey protein) [20], gut microbiota modulators (probiotics, prebiotics) [25], and camel milk [25]. However, there is no agreement on effective nutritional therapy. The mechanism underlying these therapeutics effects remain poorly understood. Of note, current clinical trials demonstrate significant methodological heterogeneity: some fail to incorporate GI symptom assessment as study endpoints, while others systematically exclude subjects with pre-existing GI symptoms, thereby limiting the generalizability of findings. As such, it is uncertain which nutritional management is optimal for GI problems in the autistic populations.

### Clinical evidence on nutritional modulation of both GI and autistic symptoms

Although the therapeutic potential of nutritional modulation for GI symptoms in ASD is clinically recognized, rigorous assessments of their dual effects on core autistic manifestations and GI symptoms remain lacking in controlled intervention studies. Thus, to provide a comprehensive perspective, we incorporated preliminary evidence from a single case report and studies that lacked strict randomization/blinding designs (Table 2).

#### Restrictive/elimination diets or structured diet plans

Dietary elimination strategies may offer therapeutic benefits for ASD by reducing gastrointestinal distress and behavioral symptoms while optimizing nutritional status. However, current evidence regarding GF/CF diets demonstrates inconsistent efficacy in ameliorating GI symptoms across ASD populations [26–28]. The low-FODMAP diet shows promise, with Nogay et al. [19] reporting significant improvements in global GI health and behavioral metrics (particularly hyperactivity and compliance), albeit without measurable effects on stool patterns. Similarly, the specific carbohydrate diet (SCD)—theoretically targeting carbohydrate malabsorption and microbial dysbiosis—has been employed to relieve GI symptoms in ASD management [29, 30]. Nevertheless, the extant evidence is limited to small-scale case reports, underscoring the need for rigorous controlled trials to establish clinical efficacy.

#### Dietary supplements

The potential role of vitamin D in addressing ASD symptoms is gaining significant attention in scientific research [20, 31], yet conclusive clinical evidence regarding its effectiveness for GI and core neurobehavioral symptom management remains insufficient. In some cases, this knowledge gap is trapped by the exclusion of ASD subjects with GI comorbidities from interventional trials, limiting the generalizability of the findings [32]. The randomized controlled trial (RCT) by Mazahery et al. [33] while demonstrating the feasibility of combined vitamin D and n-3 PUFA supplementation in ASD, found no significant improvements in GI symptoms.

Compared to single-nutrient supplementation, integrative interventions combining various nutrients and elimination diets yield superior clinical outcomes in ASD management [34]. A comprehensive follow-up survey on treatment effectiveness for ASD revealed that vitamin/mineral supplements offered the most significant benefits for both autistic and GI symptoms with fewer adverse effects [14], consistent with the earlier multimodal nutritional RCT study the author referred to [34]. Notably, a nutritional survey from the United State (*n* = 1286) revealed that magnesium and vitamin C supplementation alleviated constipation in 27% and 12% of cases, respectively [15]. Both interventions demonstrated superior benefit scores relative to conventional psychiatric and seizure medications [15].

Notably, El-Meidany et al. first established the efficacy of virgin coconut oil in managing GI symptoms in autistic children [35, 36], with their follow-up RCT confirming beneficial effects on eating behaviors [37].

Moreover, GI dysfunction in ASD may stem from impaired carbohydrate digestion, primarily due to intestinal deficiencies in glycoside hydrolase and polysaccharide lyase enzymes [10, 38]. This deficiency leads to incomplete saccharide breakdown that can trigger osmotic diarrhea, bloating, and flatulence through microbial fermentation. Therefore, administration of digestive enzymes (1.6 g papain and 0.8 g pepsin) yielded significant benefits in autistic subjects, with the treatment group exhibiting amelioration of core ASD symptoms (emotional regulation, repetitive behaviors), improved stool consistency, and decreased abdominal pain [39].

Intriguingly, research has recently uncovered the therapeutic potential of sulforaphane, a bioactive compound from cruciferous vegetables, in addressing autistic symptoms through its potent antioxidant property [20, 40]. A pioneering 12-week open-label study broke new ground by showing significant improvements in social responsiveness alongside favorable changes in urinary metabolites related to gut homeostasis in autistic children intervened with sulforaphane [41]. While these findings are promising, the current evidence lacks comprehensive evaluations on both behavioral symptoms and GI comorbidities in ASD. Similarly, several other dietary interventions with therapeutic potential for managing ASD, including phytochemicals (flavonoids, cannabinoids, and curcuminoids) [7], camel milk [25], and structured meal plans like the Feingold and Mediterranean diets [10], require more rigorous, multidimensional clinical investigations.

#### Gut microbiome-based dietary approach

Diet and gut microbiota interactions have a significant impact on the development of GI symptoms, which have also been linked to ASD mechanisms [42]. Nutraceuticals with prebiotic functions have the potential to modulate microbial homeostasis. For instance, probiotics and fructo-oligosaccharide (FOS) could work together to reduce ASD abnormalities and regulate serotonin levels [43]. In a similar vein, a 90-day supplementation with 1,3–1,6 $\beta$-glucan was shown to significantly reduce plasma α-synuclein levels, improving ASD-related symptoms and gut dysbiosis. However, this study did not assess GI conditions despite $\beta$-glucan’s known gut-modulatory effects [44].

Soluble fiber, a well-characterized prebiotic, is believed to nourish beneficial gut bacteria while alleviating constipation and symptoms associated with ASD [45]. Supporting this, a 2-month supplementation of partially hydrolyzed guar gum (PHGG) (6 g/day) markedly relieved constipation and behavioral irritability in autistic children, concurrent with reductions in serum proinflammatory cytokines [45]. Nevertheless, caution is warranted due to the absence of an established recommendation for fiber intake and the risk of side effects from overconsumption [46].

Emerging evidence indicated the role of *Candida spp*. in immune dysregulation, behavioral abnormalities, and altered brain activity, supported by its observed higher prevalence in the stool of individuals with ASD [47]. *Candida spp*. may contribute to hyperserotonemia by enhancing peripheral serotonin production while impeding brain serotonin synthesis from tryptophan, thereby exacerbating neurobehavioral symptoms (Table 1). Notably, autistic children often exhibit selective eating patterns characterized by a preference for starchy, fatty, processed, and high-simple carbohydrate foods, coupled with low protein intake [47, 48]. These dietary tendencies have prompted interest in anti-Candida dietary intervention—such as the elimination of added sugars, refined carbohydrates, and cured meats—as a strategy to alleviate both GI disturbances and autistic symptoms [49]. In addition, while a tryptophan-enriched diet has been proposed to counteract microbiome-mediated tryptophan overconsumption and hyperserotonemia, robust clinical evidence supporting its efficacy remains insufficient to justify widespread recommendation [47, 49].

**Table 1:** Clinical evidence on the effects of dietary factors on autism spectrum disorder (ASD) and gastrointestinal (GI) health

Method	Study design	Measurements	Results	Summary	References
Restrictive/elimination diets or structured diet plans					
RCT	*N* = 80; 4–16 years; GFD for 6 weeks; Iran	ROME Ш for assessing GI, GARS	Significant decreased prevalence of GI symptoms (40.57% vs. 17.10%) and improved score in behavioral disorders (80.03 ± 14.07 vs. 75.82 ± 15.37)	✔ ✔	[26]
Single-blinded trial	*N* = 66; 3–5 years; GFD for at least 8 week; Poland	ADOS-2 RRB domain, SCQ, ASRS	Improved autistic behaviors and social communication, but no changes in abdominal pain and constipation	✔ ○	[27]
RCT crossover study	*N* = 32 (25M/3F); 3–18 years; GF/CF diet for 6 months, followed by a 6-month crossover; Spain	ATEC, ERC-III, ABC, GI report	No significant changes in all the symptoms and GI issues	○ ○	[28]
RCT	*N* = 15(10M/5F); 6–17 years; low-FODMAP diet for 2 weeks; Turkey	ABC, PedsQL, Bristol stool scale	Improved GI symptoms total score and hyperactivity/noncompliance score, but no change in stool output	✔ ✔	[19]
Case study	12-year-old boy; SCD for 6 months; Canada	ATEC, GI reports	Improved GI symptoms, behavioral phenotypes, and nutritional status	✔ ✔	[29]
Case study	12-year-old boy with ASD and Fragile X Syndrome; SCD for 4 months; United States	PDDBI, GI reports, biochemical measures	Improved GI symptoms, behavioral phenotypes, nutritional status, blood markers, and sleep problems	✔ ✔	[30]
Dietary supplements					
RCT	*N* = 117; 2.5–8 years; vitamin D and/or n-3 PUFA for 12 months; New Zealand	SRS, SPM, GI questionnaire	Improved some core autistic symptoms, but no changes in GI symptoms after treatment	✔ ○	[33]
RCT	*N* = 20 (18M/2F); 3–8 years; multivitamn/mineral supplement for 3 months; United States	PGIA	Improved GI problems, sleep quality, and plasma vitamin B6, but no significant behavioral changes	○ ✔	[50]
RCT	*N* = 67 (55M/12F); 3–58 years; integrative dietary intervention for 12 months; United States	6-GSI, PGIA, ATEC, PDDBI, SRS, SSP, ABC	Improved autistic symptoms, GI symptoms total score, and constipation	✔ ✔	[34]
Open-label survey	*N* = 161 (138M/23F); ANRC-Essentials Plus for at least 3 months; United States	PGIA, NSTEA	Improved autistic symptoms, enhanced PGIA score, including GI symptoms, and a positive correlation between NSTEA scores and treatment duration	✔ ✔	[14]
Single-arm trial	*N* = 61(M49/12F); < 18 years; virgin coconut oil for 90 days; Canada	6-GSI, ATEC	Improved core autistic symptoms and GI symptoms	✔ ✔	[35, 36]
RCT	*N* = 101 (82M/19F); 3–9 years; digestive enzymes in syrup daily for 3 months; Egypt	CARS, GBRS	Improved emotional response, PGIA score, general behaviors, RRB, and GI symptoms (stool quality and abdominal pain)	✔ ✔	[39]
Gut microbiome-based dietary approach					
RCT	*N* = 41 (31 M/10F); 4–11 years; URD and/or B-GOS for 6 weeks; Britain	GI symptoms, stool consistency, ATEC, AQ, SCAS, fecal sequencing	Improved anti-social behavior and increased abundance of *Lachnospiraceae*, but no changes in GI symptoms	✔ ○	[51]
RCT crossover study	*N* = 8 (7M/1F); 2–11 years; probiotic and BCP for 5 weeks, followed by crossover to BCP alone for 5 weeks; USA	ABC, RBS, ABAS, GI symptoms questionnaires	Improved GI symptoms and autistic behaviors	✔ ✔	[52]
RCT	*N* = 26; 3–9 years; probiotics + FOS for 108 days; China	ATEC, 6-GSI, plasma metabolites, fecal sequencing	Improved autistic symptoms, GSI total score and subscales in constipation, diarrhea and stool smell, and decreased serotonin level	✔ ✔	[43]
Single-arm trial	*N* = 13 (12M/1F); 4–9 y; PHGG (6 g/day) for 2 months; Japan	ABC, defecation report, serum cytokine measures, fecal sequencing	Improved behavioral irritability, constipation, and gut dysbiosis	✔ ✔	[45]

The table summarizes nutritional intervention studies that systematically evaluated both core ASD symptoms (primary outcome) and gastrointestinal manifestations (secondary outcome). In the penultimate “Summary” column, the symbols “✔” and “○” denote statistically significant and non-significant improvements in symptoms, respectively

*RCT* randomized controlled trial, *GFD* gluten-free diet, *GARS* Gilliam autism rating scale, *ADOS-2* autism diagnostic observation schedule-second edition, *RRB* restricted and repetitive behaviors, *SCQ* social communication questionnaire, *ASRS* autism spectrum rating scale, *M* male, *F* female (unremarked if not stated in the paper), *GF/CF* gluten-free/casein-free diet, *ATEC* autism treatment evaluation checklist, *ERC-III* behavioral evaluation resumé du comportment in French, *ABC* aberrant behavior checklist, *PedsQL* pediatric quality of life inventory, *SCD* specific carbohydrate diets**,** *PDDBI* pervasive developmental disorders behavior inventory, *PUFA* polyunsaturated fatty acids, *SRS* social responsiveness scale, *SPM* sensory processing measure, *6-GSI* 6-item gastrointestinal severity index, *PGIA* parent global impressions of autism, *SSP* short sensory profile, *NSTEA* overall benefit/adverse effect scale of the national survey on treatment effectiveness for autism, *PHGG* partially hydrolyzed guar gum, *CARS* childhood autism rating scale, *GBRS* global behavior rating scales, *URD* unrestricted diet, *B-GOS* Bimuno® galacto-oligosaccharide, *AQ* autism spectrum quotient, *SCAS* Spence’s children anxiety scale, *BCP* bovine colostrum product, *RBS* repetitive behavior scale, *ABAS* adaptive behavior assessment system, *FOS* fructo-oligosaccharide

## Mechanistic insights into the influence of nutrition on GI health of ASD

The well-established bidirectional communication between the gut and brain underscores the critical importance of maintaining gut homeostasis for optimal neurological function [8, 53]. This gut–brain axis has emerged as a key focus in mechanistic research investigating how nutritional interventions may ameliorate GI disturbances in ASD [20]. Importantly, the considerable heterogeneity in ASD manifestations and the high prevalence of comorbid GI symptoms, as previously indicated, necessitate a precision approach to nutritional therapy that takes into account the underlying pathophysiological mechanisms.

### Opioid excess theory and gut barrier dysfunction

The GF/CF diet, currently the most widely implemented dietary intervention for ASD, is rooted in the “opioid excess theory” [54, 55]. This theoretical framework proposes that dietary-derived opioid peptide may modulate opioid receptor-mediated signaling pathways [54, 55]. The “opioid excess theory,” which posits that dietary-derived opioid peptides like gluten and casein can induce gut dysbiosis, increase intestinal permeability, and allows bacterial translocation, has gained increasing attention regarding the role of the opioid system in the development of ASD and gut barrier dysfunction [55]. More importantly, a substantial subset of the ASD population exhibits both compromised GI conditions and sensitivity to casein and gluten [3, 5]. This vulnerability can lead to opioid-induced inflammation and impair central nervous system functions by activating opioid receptors, particularly through dysregulation of the morphine-serotonin system [54, 56]. These collective findings indicate that the therapeutic application of GF/CF diets appears context-dependent and may not be universally implemented in ASD, with clinical benefits most pronounced in cases where GI comorbidities are present [57]. The pathophysiological rationale derives from the well-characterized intestinal barrier dysfunction observed in ASD populations, wherein a FODMAP diet may confer therapeutic benefits through two principal mechanisms: (1) downregulation of toll-like receptor 4 (TLR4)-mediated proinflammatory signaling cascades, and (2) restoration of intestinal mucosal barrier function [58]. This therapeutic approach is particularly warranted given that excessive FODMAP ingestion exacerbates intestinal hyperpermeability, potentially inducing colonic epithelial injury and subsequent low-grade inflammation [7]. On the other hand, to prevent the luminal accumulation of malabsorbed FODMAP in the small intestine, adjunctive therapy with exogenous carbohydrate-digesting enzymes may also mitigate symptoms [39, 59]. The clinical rationale for this approach is drawn from the therapeutic frameworks for IBD and irritable bowel syndrome (IBS) [2, 60, 61], supporting its potential translation to ASD treatment paradigms.

Beyond compromising gut barrier function and promoting systemic inflammation, dietary-derived opioid peptides have been shown in vitro to adversely affect neurogenesis by disrupting redox homeostasis and altering DNA methylation processes [62]. Within this context, camel milk provides two therapeutic benefits: (1) lower casein content than cow milk and (2) a rich micronutrient profile that includes antioxidants such as glutathione and superoxide dismutase [20]. These complementary benefits position camel milk as a particularly promising dietary intervention for ASD management.

### Microbiome-targeted strategies: prebiotics, probiotics, and microbial metabolites

The therapeutic efficacy of specialized diets (SCD, FOS, *β*-glucan, and PHGG) stems from their multimodal modulation of the gut–brain axis, exhibiting mechanistically similar properties to probiotic interventions [11]. In brief, dietary components with prebiotic function orchestrate microbial ecology by selectively promoting beneficial taxa (such as *Bifidobacteria* and *Lactobacilli*) [63] while suppressing detrimental species (such as *Firmicutes, Desulfovibrio,* and *Candida*) [47, 64]. This ecological shift fosters optimal SCFA production (particularly propionate homeostasis), normalization of 5-hydroxy tryptamine (5-HT) metabolism, and even involving in mitochondrial function and epigenetic regulation [65]. Importantly, the cross-talk between gut and brain can form a self-perpetuating cycle, wherein gut dysbiosis drives tryptophan depletion through both impaired absorption and aberrant 5-HT metabolism, subsequently disrupting vagal-mediated gut motility and central nervous system function [4, 8, 10]. Therefore, to achieve gut homeostasis and a balance of 5-HT and SCFA, autistic subjects experiencing GI symptoms may benefit from nutraceuticals with prebiotic properties or microbiome-targeted diets, such as a tryptophan-enriched diet or an anti-Candida diet.

### The gut–immune–brain triad as a target for nutritional approach

The gut–immune–brain triad is pivotal in the development of ASD [66]. Nutritional interventions designed to enhance GI health and neuronal function involve a complex interplay that extends beyond the gut microbiome to include dynamic cross-talk with immune responses[61, 66, 67]. Comparative transcriptomic analyses have revealed convergent inflammatory signatures between ASD and IBD, characterized by elevated zonulin levels and upregulated mucosal cytokines, indicative of impaired gut barrier integrity in ASD populations [61]. Recent evidence implicates IL-17A—a Th17-derived proinflammatory cytokine—in ASD pathogenesis, given its dual role in disrupting blood–brain barrier integrity and amplifying neuroinflammatory cascades. This positions IL-17A as a critical therapeutic target for gut–immune–brain axis modulation [68, 69]. Notably, the neuroinflammation driven by this cytokine may be counteracted by specific dietary components. Dietary polyunsaturated fatty acids (PUFAs) have demonstrated significant immunomodulatory potential by simultaneously suppressing proinflammatory TLR4/NF-κB signaling pathways involved in Th17 differentiation while promoting anti-inflammatory IL-10 production [23, 63]. Similarly, virgin coconut oil, which is rich in medium-chain triglycerides, exhibits dual functionality through its antimicrobial properties against Th17-inducing pathobionts and its ability to ameliorate intestinal oxidative stress [70]. In addition, vitamin D [71] and essential micronutrient complexes [72] exhibit anti-inflammatory and antioxidant effects. These nutritional factors appear to modulate gut–immune homeostasis via direct suppression of intestinal proinflammatory signaling pathways and attenuation of vagally-mediated neuro-immune activation, thereby mitigating gut–brain axis dysfunction in ASD [4, 8].

### Shared genetic architecture of ASD and GI issues, with nutrient–gene interaction

**Fig. 1:**
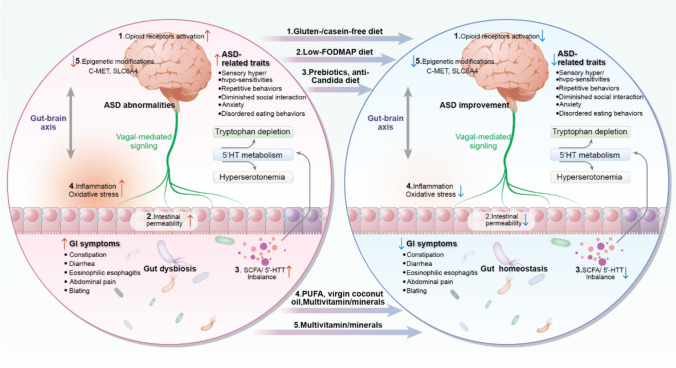
Potential mechanisms by which nutritional strategies alleviate gastrointestinal (GI) symptoms in individuals with autism spectrum disorder (ASD). The interplay between ASD-related traits and GI symptoms is characterized by disrupted gut–brain communication, leading to gut dysbiosis. Key mechanisms include: (1) over-activation of opioid receptors, (2) increased intestinal permeability, (3) imbalanced microbial metabolites resulting in tryptophan depletion and hyperserotonemia, and (4) vagally-mediated proinflammatory responses. These pathways could be tuned by specific dietary interventions. Furthermore, given the overlap in host genes such as c-MET and SLC6A4 that contribute to comorbid GI symptoms in ASD, dietary interventions may also influence the epigenetic modulation of these genes. *5-HT* 5-hydroxy tryptamine, *SCFA* short-chain fatty acids, *FODMAP* fermentable oligosaccharides, disaccharides, monosaccharides, monosaccharides, and polyols, *PUFA* polyunsaturated fatty acids

From the aspect of genetic modifications, the overlaps of genetic risk factors for both ASD and GI issues suggest a potential avenue for symptoms management through DNA methylation regulation. Vitamin deficiencies (such as D, B6, B9, and B12) observed in ASD can be mitigated with oral multivitamin supplementation [33, 57]. With adequate levels of these vitamins, lower 5-methyltetrahydrofolate levels can enhance internal methyl availability, thereby aiding in maintaining essential methyl donors [57]. For example, genetic polymorphisms in c-MET and SLC6A4, which link to serotonin transporter hyperfunction in both the brain and GI tract and disrupt the MET tyrosine kinase, are notably prevalent in autistic individuals comorbid with GI symptoms [4]. Prior evidence indicated therapeutic potential through inhibiting genes expressions and methylation alteration modified by environmental factors [73–75]. Thus, exploring epigenetic changes in these genes associated with the methylation cycle, influenced by multivitamin supplementation, opens up a new research avenue (Fig. 1).

## Nutritional pitfalls and oversights regarding GI health in ASD

### Side effects of ASD-related dietary factors on GI health

Dietary interventions have garnered considerable enthusiasm as a potential therapeutic strategy for ASD [16, 63]. While certain diets may improve neurobehavioral outcomes, they may simultaneously exacerbate GI dysfunction—a particularly concerning finding given the high prevalence of pre-existing eating disorders in this population [63].

The GF/CF diet has demonstrated potential for ameliorating behavioral symptoms in ASD, yet mounting evidence suggests it may simultaneously compromise GI health [20]. This dietary regimen often leads to insufficient fiber intake from grain sources, which may exacerbate pre-existing constipation associated with gut dysbiosis in individuals with ASD [76]. Kekker et al. [22] also provided concerning epidemiological evidence, demonstrating the GF/CF diet carried a relative risk of 2.33 (95% confidence interval [Cl] 0.69–7.90) for inducing GI discomfort. Similarly, although a low-FODMAP diet may help alleviate gastrointestinal symptoms, its restrictive nature may deplete beneficial *Bifidobacteria* and worsen constipation—a particularly problematic effect given the high frequency of chronic constipation in ASD [77]. Paradoxically, FOS, while classified as FODMAP due to their rapid fermentation and osmotic effects, have also shown therapeutic potential in improving gastrointestinal health and alleviating symptoms associated with ASD [43]. Drawing insights from their cautious application in individuals with inflammatory bowel conditions [78, 79], a low-FODMAP approach that carefully balances GI symptom management with the benefit of prebiotic appears essential for autistic subjects. Moreover, as previously noted, the biochemical rationale for KGD in ASD remains uncertain, and its association with worsened constipation, reflux, and disordered eating behaviors limits its clinical utility [21].

Oral multivitamin supplementation, particularly vitamin B12, has demonstrated therapeutic potential for ameliorating core and comorbid symptoms in ASD [80]. While the oral administration route presents convenient, it is associated with a higher incidence of GI adverse effects (though < 5% incidence) compared to B12 injection [80]. Similarly, integrative nutritional interventions involving iron, carnitine, or digestive enzymes supplementations have been linked to GI discomfort in susceptible individuals [15, 34].

In addition to dietary intervention, ASD-specific dietary preference can precipitate nutritional deficiencies with significant GI implications. The high prevalence of vitamin D and A deficiencies in ASD [10] may exacerbate gut barrier dysfunction and immune dysregulation, potentially contributing to the development of celiac disease and IBD [81]. Furthermore, as indicated by Cheng et al. [82], a mechanistic link between vitamin A deficiency and GI dysfunction in ASD could be mediated through impaired retinal dehydrogenase activity and other psychopathological factors. Therefore, prioritizing these vitamin deficiencies to constitute therapeutic strategy is essential when addressing GI dysfunction in ASD.

Autistic individuals with sensory hypersensitivity—defined by above-average scores on the Sensory Sensitivity quadrant—exhibit significantly higher fiber intake [83]. While dietary fiber is widely recognized for alleviating constipation, a prevalent GI symptom in ASD, hypersensitive individuals may experience GI distress from excessive complex carbohydrates, manifesting as bloating and flatulence [46]. Importantly, poor dietary quality has been independently associated with both GI symptom severity and core autism features [84], underscoring the necessity of systematic dietary monitoring in individuals with GI dysfunction.

Another dietary concern is the established association between ASD and increased consumption of ultra-processed foods (UPFs), which are laden with additives designed to enhance palatability and texture [7, 12]. Although no meta-analysis has directly evaluated the impact of food additives on GI dysfunction in ASD [85], epidemiological evidence links higher UPF intake to a heightened risk of Crohn’s disease (CD) (hazard ratio [HR] = 1.71, 95% CI 1.37–2.14) [86], and IBS (odd ratio [OR] = 1.25, 95% CI 1.12–1.39) [87]. Collectively, these findings highlight that dietary influences on GI health in ASD are not limited to intervention effectiveness but are inherently intertwined with the high prevalence of suboptimal eating behaviors and nutrient imbalances (Table 2). A precision nutrition framework—prioritizing personalized assessments of food preferences, nutrient status, and gastrointestinal responsiveness—becomes essential to mitigate adverse effects and maximize therapeutic benefits, aligning with the overarching goal of optimizing evidence-based dietary interventions for this complex population.

**Table 2:** Dietary determinants and gastrointestinal (GI) dysfunctions in autism spectrum disorder (ASD)-specific settings

Dietary determinants	ASD-specific settings	Evidence types	Study outcomes	References
GF/CF diet	Therapeutic diet	Systematic review and meta-analysis on RCTs	Constipation, aggravated GI problem in some cases; trigger GI discomfort (HR = 2.33, 95% CI 0.69–7.90)	[22, 76]
Low-FODMAP diet	Therapeutic diet	Systematic review	Decreased abundance of *Bifidobacteria*, excluding constipation-predominant patients	[77]
FOS supplementation	Therapeutic diet	RCT study	Increased functional GI issues in IBD patients	[79]
KGD diet	Therapeutic diet	Scoping review	Elevated disordered eating behaviors and incidences of constipation (12.5%), diarrhea (18.8%), and vomiting (18.8%)	[21]
Multivitamin containing B12	Nutritional supplementation	Meta-analysis on RCTs	Constipation/diarrhea 4.1% (0.0%, 14.0%) and nausea/vomiting 3.5% (0.0%, 11.0%)	[80]
Iron	Nutritional supplementation	Retrospective survey	Higher incidence of GI side effects (17% of the treatments) compared to other nutraceuticals	[15]
Carnitine or digestive enzymes	Nutritional supplementation	RCT study	Emerged GI discomfort	[34]
Insufficient vitamin D	Selective eating	Meta-analysis on observational studies	Increased incidences of diarrhea (OR = 1.79, 95% CI 1.15–2.80) and IBD (OR = 1.36, 95% CI 0.91, 2.04)	[88, 89]
Insufficient vitamin A	Selective eating or psychopathologic factors	Observational study	Decreased level of serum retinol in children with ASD and comorbid GI symptoms (0.7 ± 0.25 vs. 0.83 ± 0.29 μmol/L)	[82]
Fiber overconsumption	Sensory processing traits	Narrative review	bloating and flatulence side effects	[46]
Unbalanced dietary intake	Dietary preference/pattern	Observational study	Constipation (*β* = 0.198, 95% CI 0.023–0.374) and total GI symptoms (*β* = 0.231, 95% CI 0.063–0.400)	[84]
UPF containing additives	Dietary preference/pattern	Observational study and meta-analysis	Increased risk of CD (HR = 1.71, 95% CI 1.37–2.14) and OR of IBS (1.25, 95% CI 1.12–1.39)	[86, 87]

The dietary determinants in ASD-specific settings encompass three key facets: (1) specialized dietary interventions for symptom modulation, (2) trait-mediated nutritional status variations, and (3) context-dependent dietary preference/pattern shaped by neurodevelopmental characteristics

*GF/CF* gluten-free/casein-free, *RCT* randomized controlled trial, *HR* hazard ratio, *CI* confidence interval, *FODMAP* fermentable oligosaccharides, disaccharides, monosaccharides, and polyols, *FOS* fructo-oligosaccharides, *IBD* inflammatory bowel disease, *KGD* ketogenic diet, *OR* odd ratio, *UPF* ultra-processed foods, *CD* Crohn disease, *IBS* irritable bowel syndrome

### Algorithm for nutritional management of GI symptoms in ASD

**Fig. 2:**
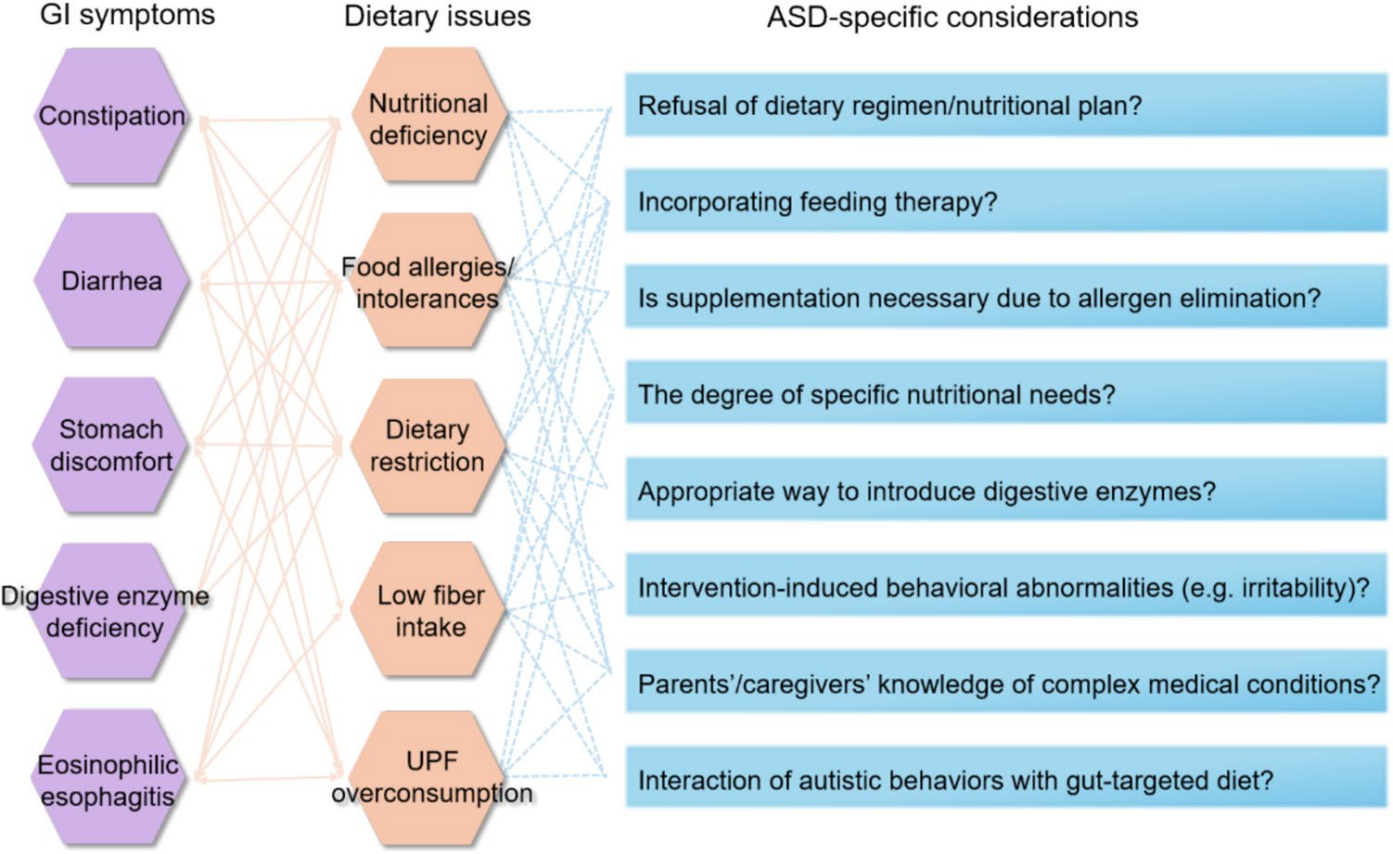
A framework for practical nutritional strategies to enhance gastrointestinal (GI) health with autism spectrum disorder (ASD)-specific considerations. Orange lines indicate existing relationships, while blue lines highlight specific considerations in the context of dietary issues. *UPF *ultra-processed foods

Despite growing recognition of GI dysfunction in ASD, standardized nutritional guidelines remain scarce, particularly given the interplay of dietary, medical, and behavioral complexities in this population. GI symptoms—ranging from chronic constipation to visceral hypersensitivity—may exacerbate selective eating behaviors and heightened disgust sensitivity, further compounding nutritional challenges in ASD. A deeper understanding of the underlying GI pathophysiology and its bidirectional relationship with ASD-specific dietary determinants is, thus, critical [48]. Although prior work has proposed a nutrition-management algorithm for GI symptoms in autistic children, its scope remains limited, addressing only two common conditions (constipation and eosinophilic esophagitis) and lacking contemporary clinical applicability [12]. Given the heterogeneity and multifactorial nature of GI disorders in pediatric ASD, individualized assessment and tailored nutritional interventions are essential. To address this gap, we propose an expanded framework (Fig. 2) built on a two-step approach: (1) identifying and addressing any obstacles that may hinder the creation of a tailored diet aimed at specific GI symptoms, and (2) ensuring that all nutrition-related concerns are thoroughly evaluated and managed through the management [12]. This framework aims to stimulate methodologically robust clinical trials, fostering targeted interventions that concurrently address GI dysfunction and neurobehavioral symptoms of ASD.

## Limitations and future perspectives

The current understanding of nutritional management in ASD-related GI disorders reveals critical research gaps marked by significant methodological variability and population heterogeneity. Existing studies demonstrate substantial inconsistencies in GI evaluations across different cohorts reported by children and their parents [2]. This heterogeneity underscores the need for multimodal evaluation strategies that integrate objective clinical measures with standardized behavioral assessments and caregiver-reported outcomes [2]. The field faces particular challenges in developing targeted interventions due to the intricate interplay between neurodevelopmental characteristics, genetic/metabolic variations affecting nutrient metabolism, and frequent misattribution of biological deficits to behavioral issues [9, 10]. As previously reported, these deficits are often misconstrued as mere eating or behavioral issues, obscuring their underlying physiological basis and impeding the development of targeted interventions [8, 9].

Furthermore, current limitations highlight pressing needs for longitudinal studies tracking nutrient status trajectories and rigorous clinical trials assessing ASD-tailored nutritional interventions addressing GI issues. In light of immature GI systems, parenteral nutrition has been commonly provided for preterm infants to improve neurodevelopmental outcomes [90], yet it is rarely considered for babies with early signs of autism and severe GI issues. Notably, parenteral supplementation with fish oils has been shown to reduce the risk of ASD and mitigate neuronal disruptions in preterm infants [91]. A recent study with the latest quantitative data has revealed the potential remission effects of partial enteral nutrition (PEN) and exclusive enteral nutrition in alleviating inflammation and GI issues [60]. Importantly, optimal micronutrient delivery should balance efficacy with tolerability. For example, subcutaneous B12 injections may circumvent GI adverse effects associated with oral supplementation in ASD populations [13]. Nonetheless, the evidence supporting enteral nutrition therapy for pediatric GI issues remains limited and its impact on ASD management requires further exploration.

Last but not least, a comprehensive approach integrating dietary and psychosocial interventions is critical for addressing ASD-related GI issues [92, 93]. Effective management requires multidisciplinary collaboration among pediatric gastroenterologists, feeding therapists, and mental health professionals with GI expertise, particularly for complex cases involving severe eating challenges.

## Conclusion

In conclusion, cross-disciplinary deficits in training, research, and publicly funded treatment for autistic individuals with comorbid eating, gut–brain, and motility disorders underscore the limitations of single-clinician approaches. Despite the potential of nutritional interventions, current clinical research falls short of high-quality trials and holistic management strategies, especially in complex medical scenarios. We propose establishing multidisciplinary care teams as the clinical standard, with dietitians playing a central role in managing the unique nutritional needs of autistic individuals. To advance evidence-based practice, key priorities include: (1) development of standardized criteria for identifying and classifying adverse food reactions, and (2) implementation of rigorous longitudinal studies to quantitatively assess dietary effects in this population. These measures are essential for developing personalized nutritional paradigms that synergistically optimize gastrointestinal health and behavioral outcomes in ASD.
